# Innervation of the Heart

**Published:** 1867-09

**Authors:** 


*Innervation of the Heart.* Translated from the *Gazette des Hopitaux*, by L. J. Frazee, M. D., Prof. of Materia Medica and Therapeutics, in the Kentucky School of Medicine.

MM. E. and M. Cyon, (of St. Petersburg,) have sent to the Academy of Sciences, a note upon the *innervation of the heart.*

During the past year, one of these experimenters, M. E. Cyon, conjointly with Charles Ludwig, had established the following facts:

1. The cardiac nerve, which commences by two roots, one from the pneumogastric, the other from the superior laryngeal, is a nerve of sensibility of the heart. It gives to the heart the power of paralyzing, by a reflex action, the tonicity of all the vessels of the organism; and it is for this reason that MM. Cyon and Ludwig have denominated it the *depressor nerve.*

2. The splanchnic nerves are the principal vascular nerves of the organism; their section reduces the pressure in the carotid to its minimum, while the irritation of their peripheral extremities doubles this pressure. The principal aim, this year, of MM. Cyon, has been to establish the fact, that, contrary to the theory of Bezold, the acceleration of the heart's beats, produced by the irritation of the spinal marrow, is not dependent upon vascular pressure. They cut the splanchnic nerves, and, though the pressure was thus reduced to the minimum, the heart's beats were not less accelerated by the electric irritation of the spinal marrow, separated from the brain at the point of the atlas.

(These experiments were made upon animals poisoned by curari, and in which artificial respiration was maintained, after the cutting of the pneumogastric, the depressor and the sympathetic nerves of the neck.)

Having thus proved the direct action of the spinal cord upon the heart, MM. Cyon extirpated all the nerves which the heart receives from the spinal marrow, through the medium of the sympathetic ganglia, (the inferior cervical, and the superior dorsal.) This extirpation produced no change either in the number or the strength of the cardiac contractions; but after this the contractions ceased to be influenced by irritation of the spinal cord.

They attempted to prove, by directly irritating the cardiac nerves, the part which each of these plays. Similar experiments made upon rabbits and dogs, gave the following results:

1. Electrical irritation of the third branch of the inferior cervical ganglion, provoked, in rabbits, an acceleration of the heart's beats, and a diminution of their extent.

2. The first two branches of the same ganglion, are nerves of sensation of the heart, and form the continuation of the depressor nerve.

3. Irritation of the fourth branch of this ganglion, which passes above the subclavian artery, and forms, with a fifth branch of the same ganglion, the ring of Vieussens, produced a slight elevation above the average pressure of the blood without changing the number of pulsations.

4. In dogs, where the sympathetic nerve of the neck and the pneumogastric, are found in the same sheath, irritation of the *second* branch of the inferior cervical ganglion, provokes the same changes that irritation of the *third* branch produces in rabbits.

The acceleration of the pulsations produced in dogs and in rabbits, by direct irritation of the nerves described, is less marked than that produced by exciting the spinal cord, which finds an easy explanation in the fact that in the latter case all the cardiac nerves are simultaneously irritated. MM. Cyon have proposed to call these branches of the cervical ganglion *accele-*

*rator nerves of the heart*; and as to the mode of action of these nerves, they have arrived at the following conclusions:

*a* These are not the ordinary motor nerves, terminating in the muscle of the heart:

1. Because their irritation produces no tetanus of the heart;
2. It does not even augment the action of the heart, for we have seen the height of the mercurial column in the *manometer* diminish while the number of beats increased;
3. The heart has, within itself, excitor ganglia;
4. The curari does not paralyze these accelerator nerves.

*b*. Neither are these nerves which act upon the vessels of the heart, for the complete occlusion of these vessels does not change the number of the heart's pulsations.

*c*. They can only be nerves terminating in the ganglia of the heart. Their action consists in modifying the labor of the heart as to time. Thus they are simply antagonists of the pneumogastric nerves, in this sense, that irritation of these last named nerves slackens the pulsations of the heart, in augmenting their extent, while the accelerator nerves augment the number of pulsations in diminishing, at the same time, their extent.

To recapitulate, it is seen that the researches of MM. Cyon complete that which was previously known in regard to the innervation of the heart. It was known that the heart, in itself, possessed a little independent nervous center, consisting of ganglia. It was known that this little nervous center acted solely in a normal and continuous manner, for the purpose of regulating the contractions of the organ. It was known that by their influence upon this little center, the pneumogastric nerves entering into action, slacken the pulsations of the heart in augmenting their extent.

MM. Cyon have ascertained that the pneumogastrics have antagonists springing from the spinal marrow, and exerting a contrary influence upon the little cardiac center. Thus the general innervation will be found doubly bound in a centrifugal way to the proper innervation of the heart.—*West. Jour. of Med.*

